# Cre-loxP-mediated genetic lineage tracing: Unraveling cell fate and origin in the developing heart

**DOI:** 10.3389/fcvm.2023.1085629

**Published:** 2023-02-27

**Authors:** Tao Wang, Xinzhe Chen, Kai Wang, Jie Ju, Xue Yu, Shaocong Wang, Cuiyun Liu, Kun Wang

**Affiliations:** Institute for Translational Medicine, The Affiliated Hospital of Qingdao University, College of Medicine, Qingdao University, Qingdao, China

**Keywords:** Cre-loxP, genetic lineage tracing, endocardial cells, epicardial cells, cardiomyocytes, cardiovascular diseases

## Abstract

The Cre-loxP-mediated genetic lineage tracing system is essential for constructing the fate mapping of single-cell progeny or cell populations. Understanding the structural hierarchy of cardiac progenitor cells facilitates unraveling cell fate and origin issues in cardiac development. Several prospective Cre-loxP-based lineage-tracing systems have been used to analyze precisely the fate determination and developmental characteristics of endocardial cells (ECs), epicardial cells, and cardiomyocytes. Therefore, emerging lineage-tracing techniques advance the study of cardiovascular-related cellular plasticity. In this review, we illustrate the principles and methods of the emerging Cre-loxP-based genetic lineage tracing technology for trajectory monitoring of distinct cell lineages in the heart. The comprehensive demonstration of the differentiation process of single-cell progeny using genetic lineage tracing technology has made outstanding contributions to cardiac development and homeostasis, providing new therapeutic strategies for tissue regeneration in congenital and cardiovascular diseases (CVDs).

## Introduction

Understanding the origin and fate of cardiac progenitor cells is of great significance for unraveling the developmental properties of main cardiac cell lineages and the pathogenesis of various cardiac diseases. The heart contains a extraordinarily complex lineage of cells, primarily cardiomyocytes and non-cardiomyocytes. Nuclear bomb test-derived ^14^C and genetic cell-lineage tracing analysis revealed that cardiomyocytes account for 25–35% of all cells in the heart, with the remainder being non-cardiomyocytes including endothelial cells (ECs), mesenchymal cells, hematopoietic stem cells, and fibroblasts (1–3). To further reveal the characteristics of cardiac cell lineages, cardiac cardiomyocyte counts were measured in 29 subjects ranging in age from 1 month to 73 years. The number of cardiomyocytes remains constant throughout human life, and the cardiomyocyte turnover rate is less than 1% per year in adult cardiomyocytes (1). In recent years, the continuous development and innovation of genetic lineage tracing technology have promoted the study of the developmental characteristics and function of cardiac cell lineages. The genetic lineage tracing tools based on cyclization recombinase (Cre) and site-specific locus of x-over, P1 (loxP) homologous recombination elucidate the characteristics of the developmental origin and fate of cells in different organs (4). Cre-loxP provides a new research tool for further revealing the regeneration, homeostasis and pathogenesis of cardiac tissue (5). The regulatory mechanisms of cell origin and fate elucidated the fate and fate plasticity of various cell progeny in humans.

With the advancement of a prospective study, the accuracy of Cre-loxP-mediated genetic lineage-tracing systems has been constantly iterated and updated. Many new genetic lineage tracking systems for different cell lineages have been developed, such as epicardial progenitors, epicardial cells, and cycling cardiomyocytes. Zhou Bin et al. (6) applied a genetic lineage tracing system to effectively monitor the development of endocardial cell progeny over a prolonged period, denying the contribution of cardiac fibroblasts (CFs) to angiogenesis. Mapping all proliferating cells and their progeny in the heart by Ki67 single-cell mRNA sequencing and genetic lineage tracing found no evidence of a quiescent cardiac stem cell population. Dual recombinases dramatically improved the precision of genetic lineage tracing and reduced pitfalls in lineage-tracing studies due to Cre expression in pre-existing non-targeted cells. The lineage tracing system based on dual-recombinase-induced cross-reporter genes is called dual-recombinase-activated lineage tracing with an interleaved reporter (DeaLT-IR) (7). This site-specific recombination at the DNA level is permanent and irreversible and is an indispensable aid in studying cardiac structural development and congenital heart disease.

CVDs remains the leading cause of death in Western countries, including myocardial infarction, myocardial hypertrophy, hypertension, and congenital diseases, which seriously threaten human life and health (8). In recent years, the American Heart Association (AHA) and the Centers for Disease Control and Prevention (CDC) have reported that mortality from CVDs has declined. Still, it remains a heavy health and economic burden for the United States and the world (9). Studies have shown that most cardiomyocytes exit the cell cycle shortly after birth and eventually become terminally differentiated cells (10, 11). The heart has minimal regenerative capacity compared to organs, such as the liver, skin, and skeletal muscles (12). Even if the heart is injured, it is difficult to re-enter the proliferative cycle. Therefore, any loss of cardiomyocytes is irreversible and may cause or exacerbate heart failure. In 2011, genetic fate mapping revealed that most cardiomyocytes in regenerating tissues were derived from pre-existing cardiomyocytes, that is, by direct division of cardiomyocytes (13). Genetic lineagetracing studies of epicardial cells have revealed several previously unexplored cardiomyocyte progenitor populations, with significant implications for cardiomyocyte origin in the interventricular septum and atrioventricular wall. Therefore, achieving selective and more precise control is a core criterion for Cre recombinase-based cell fate lineage tracing. Following cardiac injury, genetic fate mapping provides a potential therapeutic strategy and theoretical framework for vascular and myocardial tissue regeneration and repair.

## Genetic lineage tracing

In the 1960s, as the use of lineage tracing in cell development increased, the technology was constantly updated and reinvented. In the process of egg cell division, cell lineage effectively infers that the differentiation fate of specific cells in the early embryo is uncertain due to several factors (14). Using genetic lineage tracing to effectively reveal the growth pattern of high clonal potential cancer stem cells and the fate determination and proliferative potential of individual tumor cells (15). It is also an essential tool for studying the fate properties of adult mammalian tissue stem cells. Throughout the lineage tracing system, the fate of a single cell is indiscriminately passed on to offspring, resulting in a set of stable and permanently marked clones (16). It is worth noting that genetic markers will not affect the normal life activities and properties of neighboring cells. At the same time, recent advances in single-cell sequencing (17–19) to elucidate cell type composition present enormous opportunities and challenges. To enable this marker to identify and classify cells as accurately as possible, single-cell transcriptomic-based lineage tracing improves the resolution and reliability of progeny differentiation trajectories. Lineage tracing provides a powerful means of understanding the relationship between tissue regeneration, homeostasis, and disease, especially when it is cross-talked with signals that regulate cell fate decisions.

In addition to the traditional Cre-loxP system, Dre expression from the En1 (Dre)-rox recombinase system is also an essential technical means to precisely track the cell fate of some progenitor and stem cells (20, 21). However, Cre recombinase may be confounded in non-target cells and thus affect the accuracy of cell fate tracking. The Cre-loxP and Dre-rox recombinase systems carry different reporter genes, such as the ZsGreen and tdTomato reporter genes, which can eliminate the interference of non-target cells by precisely identifying specific and staggered sites (7). This was further demonstrated by the tracer study of cardiomyocytes, which were labeled and induced by crossing IR and constitutive Tnni3-Dre and αMHC-MerCreMer mice. As a result, the Tnni3-labeled cardiomyocytes were all tdTomato^+^. Thus, expression of the Dre-rox mediated recombination system inhibited tamoxifen (TAM)-induced Cre-loxP recombination in cardiomyocytes. This system of dual recombinases precisely acting on cross-reporter genes addresses untargeted and unintentional lineage tracing (22). By the same principle, using a dual histone-mediated genetic system revealed the inability of peritoneal or pleural macrophages to invade deep organs for organ regeneration during lung and liver injury (23).

## Principles and approaches of the Cre-loxp system

Cre recombinase is a 38 kDa protein encoded using P1 bacteriophage, which is highly conserved and catalytically active and can efficiently achieve site-specific DNA fragmentation and recombination both *in vivo* and *in vitro* (24–26). Similarly, the locus of crossing over in phage P1 (27, 28) is also derived from the P1 phage, composed of two 13 bp inverted repeats and an 8 bp sequence in the middle. It is a specific site for Cre recognition and recombination. According to the site orientation of the loxP sequence, Cre recombinase can efficiently excise site-specific DNA, sequence inversion, the exchange of double strands or chromosomal translocation (29, 30). The primary condition for the Cre-loxp system to recombine is that the flanking sequence of a DNA fragment to be deleted or recombined must contain 13 bp of floxed DNA sequence. Second, cells and animals carrying loxP DNA must drive the cell-specific expression of Cre recombinase, including DAG, ATCB, c-Kit, Ki67, and αMHC. Since the birth of Cre-loxP technology in 1981, as of 2015, more than 3,000 research reports have been recorded in the PubMed database (31). Consequently, the potential for the application of Cre technology is unparalleled. Cre-loxP system initially focuses on achieving the expression or deletion of target cell genes and constructing conditional gene expression and knockout mice (32, 33). The Cre recombinase-constructed mice are crossed with flox mice, and Cre accurately recognizes the specific site of the target gene to obtain transgenic or knockout mice. Notably, conditional gene expression mice showed a lox-stop-lox (LSL) sequence upstream of the target gene. When the specific Cre recombinase excises the LSL sequence, it further activates the expression of the downstream gene of interest (GOI) (34).

**Figure 1:**
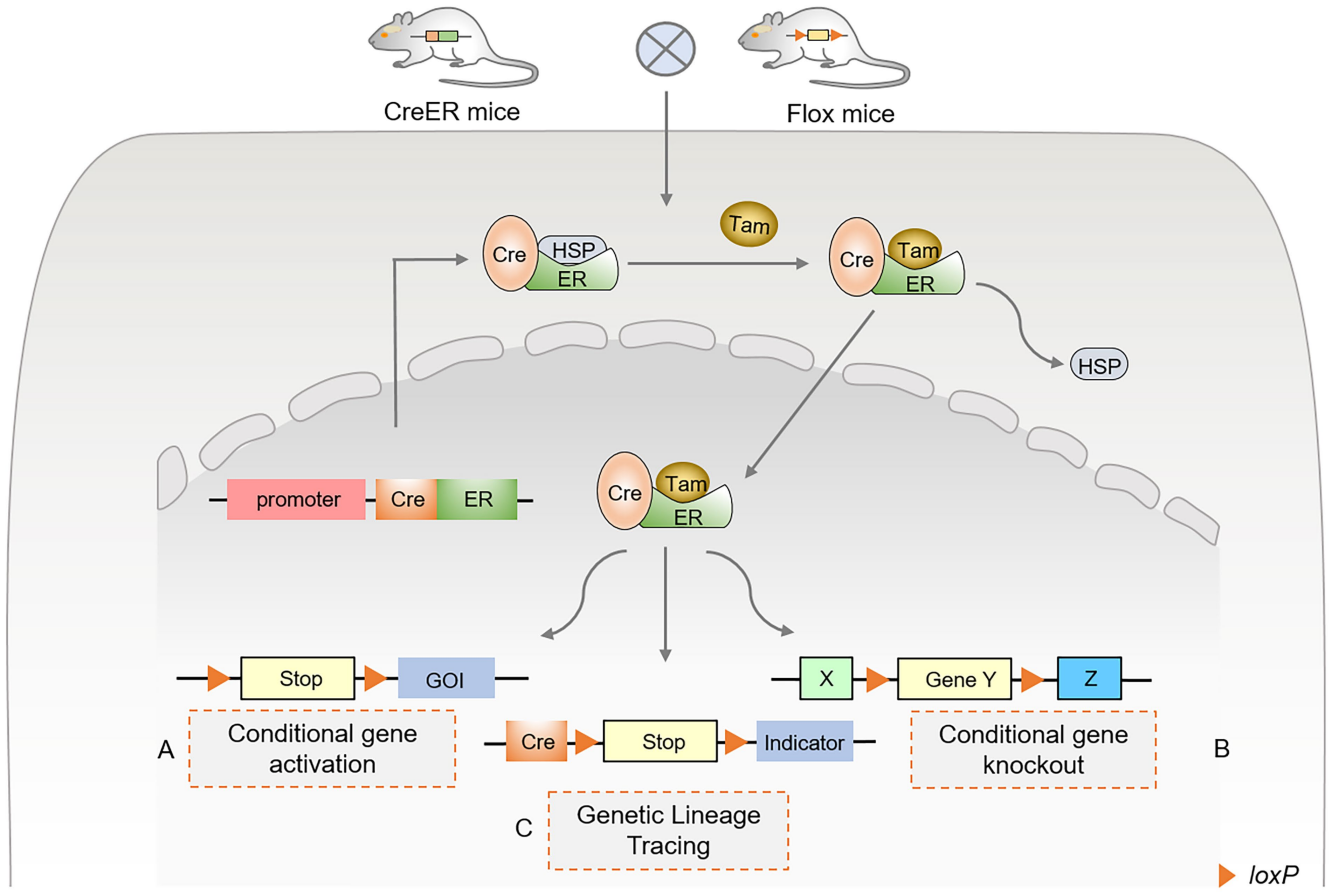
Principles and approaches of inducible Cre-loxP-mediated site-specific recombination. **(A)** Conditional gene activation. After TAM administration, Cre recombinase enters the nucleus to excise lox-stop-lox (LSL), resulting in the expression of the downstream gene of interest (GOI). **(B)** Conditional gene knockout. After TAM administration, Cre recombinase binds to the estrogen receptor (ER). It undergoes nuclear translocation, excising the loxP sequences that flank the same direction at both ends of the specific target gene. **(C)** Genetic lineage tracing using Cre-loxP. The Cre-loxP system tracks single-cell fate and origin by triggering the expression of the targeted cell indicator. The Cre-induced lox-stop-lox (LSL) sequence was recombined, and the translation stop codon was excised. Various fluorescent proteins are expressed in targeted cells as indicators or tracers, permanently marking the differentiation fate of cells.

With the development and application of Cre-loxP technology, gene regulatory functions and disease prevention of cell populations have been widely monitored in various tissues and environments. TAM binds to the estrogen receptor (ER) to activate the expression of Cre recombinase, collectively referred to as the inducible Cre-loxP system, also known as the CreER system. After TAM was injected into mice, the complex formed by CreER and heat shock protein 90 (HSP90) (35, 36) dissociated and nuclearly translocated, in turn activating the Cre-loxP system (Figure 1). In addition, several indicators were selected for specific single-cell lineage tracing, such as LacZ, GFP, red fluorescent protein (RFP), tdTomato, and Cherry. Activation of specific ERs using TAM and doxycycline (DOX) forms an inducible Cre-loxP system that can be used to study gene function at specific stages of an organism’s developmental process, mainly in the CreER and CreTet systems (4, 37, 38). The inducible Cre-loxP system improves its precision and spatiotemporal specificity, revealing gene function and cell lineage tracing studies at specific stages.

## Advances in genetic lineage tracking technology

**Figure 2:**
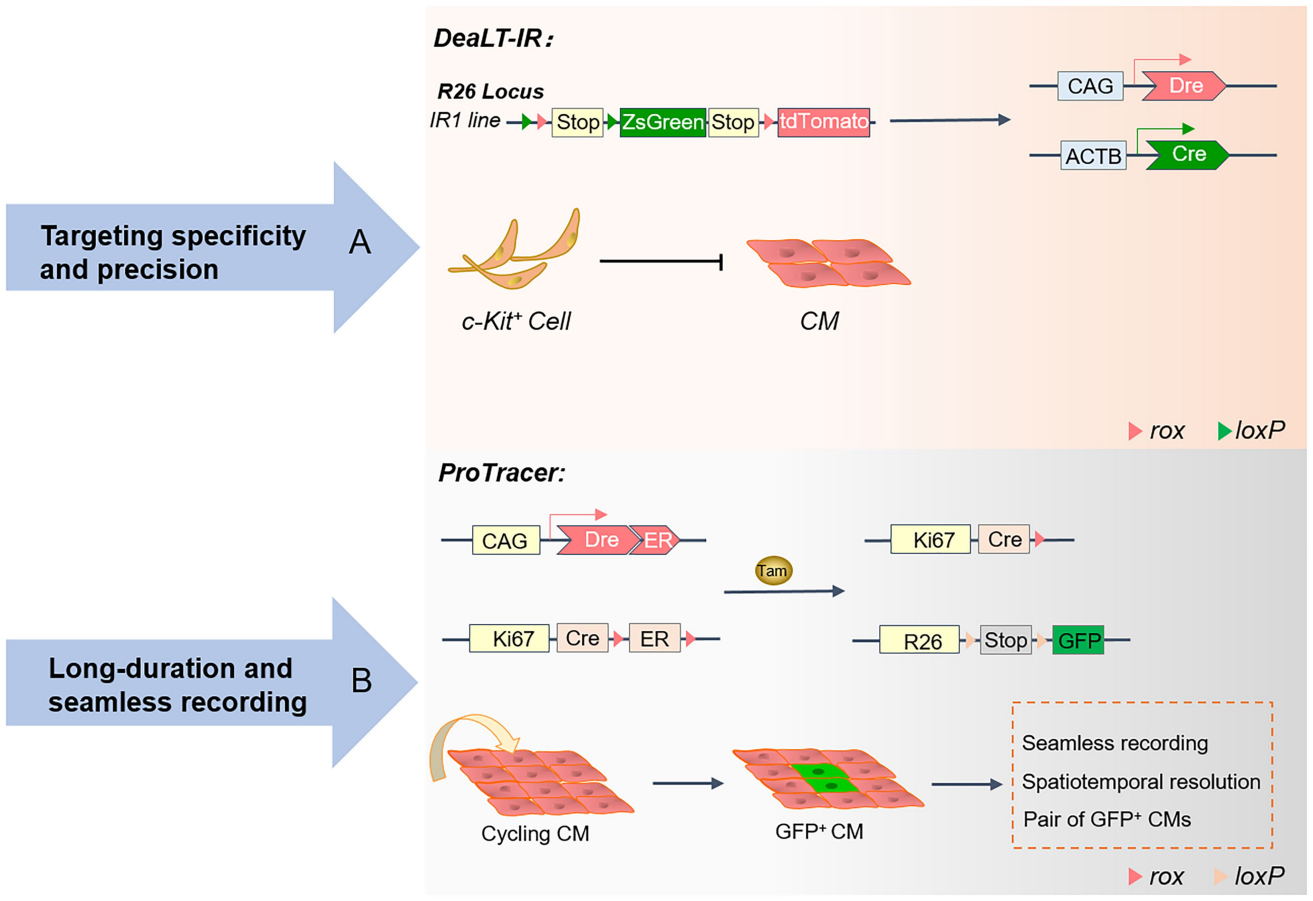
Advances in the genetic lineage-tracing system: A schematic overview of the DeaLT-IR and ProTracer systems. **(A)** The Cre-loxP and Dre-rox-based DeaLT-IR system sequentially activates the expression of the interleaved reporter (IR) genes R26-rox-tdTomato and R26-loxP-ZsGreen. In contrast to conventional lineage tracing, ZsGreen+ labeled CMs are not generated in c-Kit+ non-CMs. **(B)** Generation of the ProTracer system and identification of concentrated regions of CM cell cycle activity. The Ki67-CrexER genotype is converted to the Ki67-Cre genotype, and the reporter gene will permanently record the differentiation fate of Ki67+ cycling CM.

The Cre-loxP system (39) controls the activation or repression of tissue-specific gene function, and expression in progenitor cells generates progeny cells that are labeled by reporter genes, and this genetic marker is heritable and permanent (40, 41). To comprehensively understand the characteristics and limitations of tissue development at various levels, stages, time, and space, new genetic lineage tools based on Cre-loxP have emerged, including the DeaLT-IR system (7) and the proliferation tracer (ProTracer) system (42) (Figure 2). In recent years, the Cre-loxP system has been widely used in cardiac structural development, especially in cardiac cardiomyocyte proliferation, angiogenesis, epicardial formation, and cardiac fibroblast fate determination. Therefore, the evolving Cre-loxP is an effective research tool for studying CVDs and pathogenesis.

### The DeaLT-IR system

Hypoxia and ischemia of cardiomyocytes caused by myocardial infarction are often irreversible. The loss of cardiomyocytes eventually leads to pathological cardiac remodeling and heart failure, leading to the patient’s death (43). Understanding the active localization of cycling cardiomyocytes provides conclusive evidence of cardiomyocyte proliferation and cardiac repair (44). Although the proliferation of pre-existing cardiomyocytes in the heart is consistently recognized, the study of the regeneration of endogenous cardiomyocytes has technical limitations and challenges. In previous studies, isotope labeling (45, 46) and immunofluorescence staining (47) for proliferation markers were helpful in observing cardiomyocyte proliferation rates. At the same time, the incorporation of nucleotide analogs (38, 48) was introduced to track the proliferative potential of cardiomyocytes over time. Unfortunately, these tend to confuse cardiomyocytes with non-cardiomyocytes. Several emerging genetic lineage systems reveal cardiomyocyte fate through precise identification and spatiotemporal specificity. At the same time, these lineage-tracing techniques have contributed to the debate over the existence of embryonic stem cells (CSCs).

In the adult heart, the turnover rate of only a small fraction of cardiomyocytes typically remains between 0.45 and 1% (45). The severe deficit in cardiomyocyte proliferation makes it difficult to alleviate the loss of large numbers of cardiomyocytes during myocardial infarction, leading to heart failure and cardiac remodeling. Beltrami (49) and Ellison (50) are convinced that endogenous CSCs in the heart have the potential to differentiate into cardiomyocytes and are involved in regenerative repair and the regulatory functions of the heart. However, there has been no consensus on this statement. The study of cardiac stem cell populations has been controversial, and c-kit lineage-tracing studies have disproved this idea. The DeaLT-IR system, which consists of Cre and Dre dual recombinases and IR genes, is widely used to exclude the heterogeneous expression of Cre itself to prevent the misdirection of Cre-loxP.

In conclusion, TAM activates Kit-CreER in c-Kit^+^ non-cardiomyocytes and generates ZsGreen^+^ cardiomyocytes after cardiac injury due to cardiomyocyte differentiation of c-Kit^+^ non-cardiomyocytes rather than markers of cardiomyocytes themselves. The DeaLT-IR system precisely revealed the essential scientific question that c-Kit^+^ CSCs are not a source of cardiomyocytes. Under Cre recombinase and TAM induction, c-Kit^+^-Cre mice were crossed with Tnni3-Dre × Kit-CreER × IR mice to permanently mark the differentiation fate of cardiac progeny. The endogenous cardiac c-kit cell proliferation ratio is approximately 0.008, which is a negligible contribution to the source of new cardiomyocytes. Using three reporter gene-tagged mouse tools, including c-kit-H2B-tdTomato/+, c-kit-nlacZ-H2B-GFP/+, and c-kit-MerCreMer/+; ROSA26R-tdTomato/+, the heart-resident c-kit cells were precisely tracked and found to have only the potential to develop into ECs, rather than cardiomyocytes or smooth muscle cells (43, 51). Furthermore, no co-expression of Nkx2.5 could be observed in the progeny of kit^+^-labeled cells in the region where cardiac injury occurs. By generating the Ki67 genetic lineage tracing system, all proliferating cells in the heart and their progeny were mapped, and no evidence of a quiescent CSC population was found (3). Future research on cardiac repair and regeneration will be based on the developmental properties of ECs.

### The ProTracer system

Numerous markers of cell proliferation are critical means of studying cell cycle activity and division. Ki67 (52, 53) is a nuclear antigen of proliferating cells widely expressed in all cell cycle phases G1, S, G2, and M, except for the stationary G0 phase. The National Society of Clinical Oncology lists Ki67 as a biomarker for the routine detection of tumors and is included in the pathological evaluation of early-stage tumors (54). However, using Ki67 alone to estimate cardiomyocyte proliferation may over-calculate the proportion of proliferation. 5-ethynyl-2′-deoxyuridine (EdU) is a thymidine analog that is easily inserted into replicating DNA molecules as cells proliferate (55, 56). An efficient and rapid cell proliferation detection analysis based on the conjugate reaction of EdU and dye can effectively detect the percentage of cells in the S phase. EdU incorporation assays are commonly used to monitor the level of DNA synthesis in mouse cardiomyocytes at different developmental stages for segmented and continuous labeling.

Phosphorylated histone 3 (pH3) (57, 58) is phosphorylated on histone serine 10 and is a mitosis marker. Phosphorylation of H3 is highly conserved and occurs in specific phases and chromosomal sites in mitosis and meiosis, but the phosphorylation level is often highest in the metaphase. Aurora B is a mitotic serine/threonine protein kinase that localizes to the central spindle body during late division (59, 60). Aurora B is involved in forming the central spindle, regulates the formation of contractile rings and the cytokinesis cut-off checkpoint and is a transient structure formed during cytokinesis. However, these markers are suitable only for tracking the proliferation of cardiomyocytes at a specific moment. They are unable to continuously track the developmental fate of cardiomyocytes for a long time. The Cre-loxP-mediated genetic lineage tracing system has emerged, which continuously and accurately marks the characteristics of cell fate and will serve as a powerful helper in the study of cardiomyocyte proliferation. New technical elements are constantly enriched, which is conducive to further uncovering the underlying mechanisms of cardiac development, disease, and regeneration and repair. Genetic lineage tracing provides obviously evidence, especially during cardiomyocyte proliferation.

The Ki67-CrexER genotype was converted to the Ki67-Cre genotype under initial TAM induction by generating a cross between Ki67-CrexER and the R26-DreER mouse (42, 61). The proliferation tracer (ProTracer) (42) system permanently records GFP-tagged Ki67^+^ cells, addressing the bottleneck of the inability to continuously track and monitor cardiomyocyte cycle activity. In addition, the study showed that the efficiency of ProTracer in labeling the cardiomyocyte cycle was not significantly different from that of the isotopic method (46). Once TAM initiates the expression of DreER, Dre recombinase converts inducible Ki67-CrexER to constitutive Ki67-Cre. The ProTracer system continuously tracks single-cell progeny, enabling high-resolution, low-signal-to-noise seamless tracking of cardiomyocyte proliferation fate. The cardiomyocyte-specific ProTracer system was generated by hybridizing the cardiomyocyte-targeting virus AAV9-Dre with Ki67-CreER and R26-GFP mice. AAV9 is a myocardial-targeting virus that specifically infects cardiomyocytes. Therefore, the researchers used cardiomyocyte-targeting viruses to uniquely initiate the ProTracer recording system in cardiomyocytes. The system excludes the interference of a large number of non-cardiomyocytes. Immunofluorescence staining showed that 80% of GFP^+^ cardiomyocytes were labeled near the heart’s left ventricle (42). Consistent with previous studies, increased myocardial cell cycle activity was tracked in the myocardial infarct region using the ProTracer system. The ProTracer system precisely records the division activity of adjacent paired GFP^+^ cardiomyocytes through sparse labeling experiments.

### Fate and origin of newly formed cardiomyocytes

The Cre-loxP-mediated genetic lineage tracing system uncovers cardiomyocyte fate-determining mechanisms in cardiac development, disease, and regeneration. Researchers have demonstrated that cardiac regeneration can be triggered after apexectomy in neonatal mice. The origin and fate differentiation of cardiomyocytes were further examined using genetic lineage tracing. Rosa26-LacZ reporter mice were crossed with αMHC-MerCreMer mice (13). Most of the newly formed cardiomyocytes in the apex were labeled with LacZ, which verified that the newly formed cardiomyocytes were mainly derived from existing cardiomyocytes.

American scientists have identified four cell cycle regulators, CDK1, CCNB, CDK4 and CCND (62), which achieve stable cytokinesis by jointly activating the cell cycle. Due to potential errors in using EdU and PH3 as markers for DNA synthesis and cytokinesis, a mosaic analysis with double markers (MADM) (63, 64) lineage tracing system was employed. The cell-specifically expressed Cre recombinase was recombined with the a-MHC-MER-CRE-MER MADM cell line. A single fluorescent protein clearly indicated that the cells had undergone division. Cre recombinase-based lineage tracing revealed that up to 15–20% of cells in the field proliferated after myocardial infarction (62). Additionally, the cardiomyocyte differentiation fate of site-specific knockout of Hoxb13*^fl/fl^* mice was monitored by crossing Myh6mERcremER and MADM mice with Hoxb13 mice (48). Loss of Hoxb13 initiates the cardiomyocyte cycle, prolongs the window of cardiomyocyte proliferation, and improves pathological remodeling and fibrosis after myocardial infarction.

Low-density lipoprotein receptor-related protein 6 (LRP6) is indispensable in the canonical myocardial proliferation signaling pathway Wnt/β-catenin (65, 66). Alleles and transgenic mouse lines for LRP6 were quickly established. α-MHC-MerCreMer-tdTomato genetic lineage tracing system is composed of α-MHC-MerCreMer(Cre^+/−^) mice, LRP6^Flox/Flox^ mice and Rosa26-tdTomato mice (47). To continuously track the cardiomyocyte lineage after Lrp6 knockout, αMHC-Cre mice were crossed with Rosa26-tdTomato mice, and immunofluorescence showed that the Lrp6 knockout progeny exhibited strong proliferative ability. Understanding the basic biological developmental characteristics of adult cardiomyocytes (ACMs) is beneficial for supplementing the formation of new cardiomyocytes in the myocardial infarction area and minimizing myocardial remodeling and scarring. Hybridization between β-Actin-green fluorescent protein transgenic mice and Myh6-MerCreMer-tdTomato/LacZ mice was used to continuously track the division process of adult cardiomyocytes (67). Mature ACMs can re-enter the cell cycle and form new cardiomyocytes in three steps: (1) dedifferentiation, (2) proliferation, and (3) redifferentiation.

As early as 2015, based on Cre recombinase-induced lineage tracing, ERBB2 (68, 69) activated the myocardial cycle and proliferation to trigger mammalian cardiac regeneration. It is well known that Hippo-YAP (70, 71) is a canonical signaling pathway for cardiac regeneration. Understanding the interaction mechanism between the ERBB2 and the canonical Hippo-YAP signaling pathway is effective for cardiac regeneration therapy strategies after myocardial infarction. αMHC-MerCreMer mice (72) were crossed with YAP flox and ROSA26 tdTomato mice (73) to label the cardiomyocyte lineage with tdTomato fluorescent protein. Studies have shown that ERBB2-driven myocardial proliferation promotes the epithelial-mesenchymal transition (EMT) process and the activation of ERK-dependent YAP phosphorylation at S274 and S352 (74).

Using the same transgenic mouse lines, the αMHC-MerCreMer and R26-tdTomato mice, further expanded our knowledge of cardiac regeneration. When cardiomyocytes were exposed to 7% oxygen, TAM-induced tdTomato^+^ cardiomyocytes were heavily labeled (75). This research pioneered new insights into the role of moderate hypoxia in cardiac regenerative medicine. To investigate the role of enzymes related to glycolytic metabolism in cardiac regeneration, pyruvate kinase muscle isozyme 2 (Pkm2) (76) came to the attention of researchers. CM-specific Pkm2 modified mRNA (_CMS_Pkm2 modRNA) was used to study the contribution of strong Pkm2 expression in the cardiomyocyte cycle and proliferation. _CMS_modRNAs and Rosa26^mTmG^ mouse strains were used as models for lineage tracing, and CMSPkm2 or _CMS_Luc were finally crossed with _CMS_Cre modRNA mice to generate GFP permanently labeled cardiomyocytes (77). Thus, studies have demonstrated that Pkm2 is an essential positive regulator of the myocardial cell cycle and reduces oxidative stress damage. The application of genetic lineage tracing provides us with a precise concept.

## The developmental fate of epicardial progenitors

**Figure 3:**
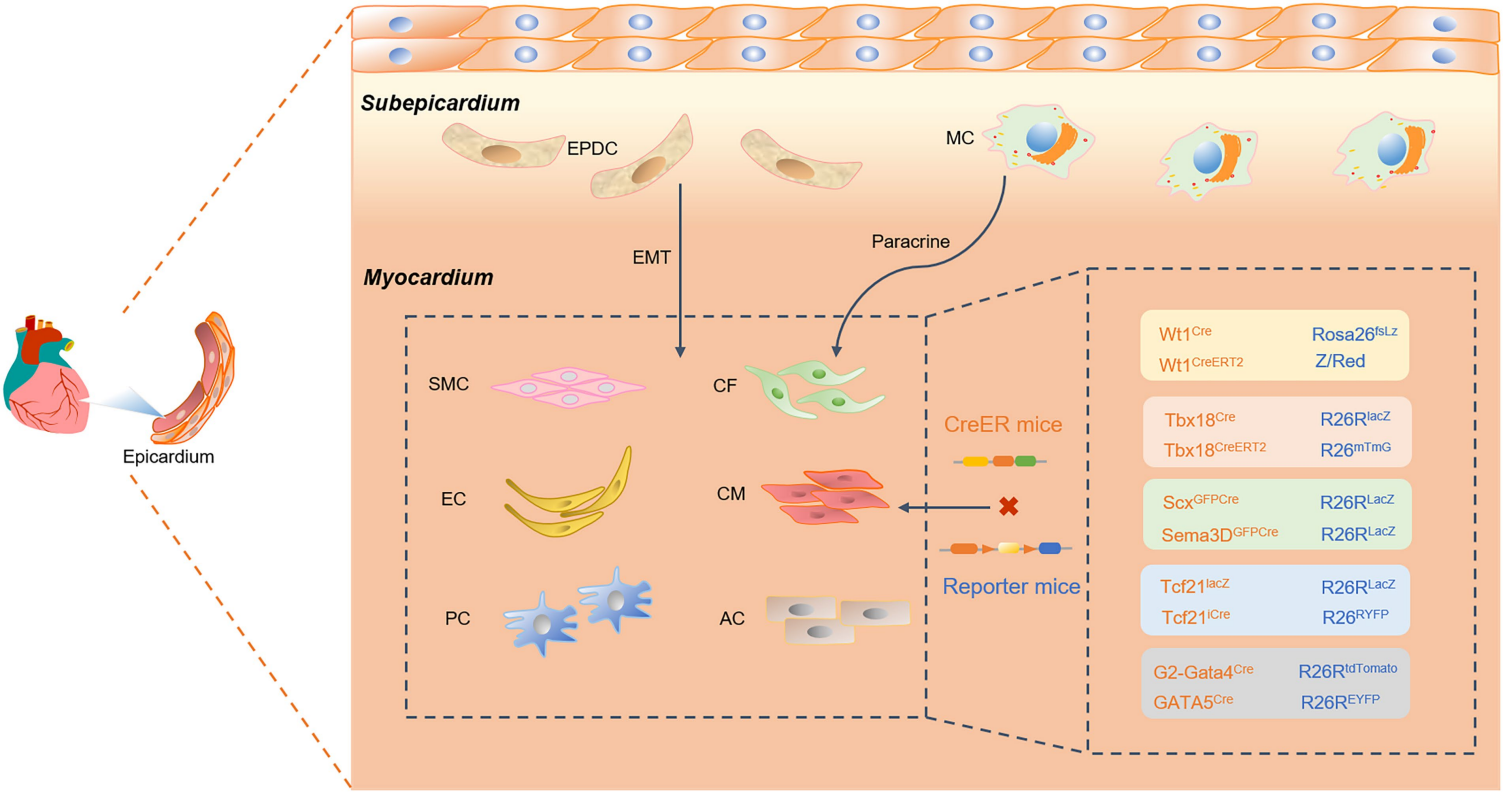
A Cre-loxP-mediated genetic lineage-tracing system was used to track epicardial fate differentiation. Epicardial-derived progenitor cells (EDPCs) are a reliable source of vascular smooth muscle cells (SMCs), pericytes (PCs), and cardiac fibroblasts (CFs) through the transformation of epithelial cells into mesenchymal (EMT) cells. Specifically expressing epicardial Cre transcription factors, such as Wt1, Tbx18, Scx and Sema3D, TCF21, and GATA4/5, the epicardium can also generate endothelial cells, cardiomyocytes, and ACs. Cre recombinase specifically labels epicardial transcription factors such as Wt1, Tbx18, Scx and Sema3D, Tcf21, and GATA4/5. This study found that the epicardium can generate endothelial cells (ECs), cardiomyocytes (CMs), and ACs. In addition, the epicardium recruits macrophages (MCs) and promotes CM proliferation through paracrine signaling.

The epicardium is a layer of mesothelial tissue covering the surface of the heart, and epicardium-derived progenitor cells with EMT properties (78–80). Epicardial-derived progenitor cells (EDPCs) are an essential source of vascular smooth muscle cells (SMCs), pericytes (PCs), and CFs (Figure 3). In addition, transcription factors released by paracrine in the epicardium further trigger cardiomyocyte proliferation and cardiac regeneration. Therefore, using a genetic lineage tracing system to explore the differentiation fate of epicardial cells has prospective implications for cardiac development and regenerative repair. Numerous studies have demonstrated that epicardial cells provide nutritional and structural support for cardiac development and adult cardiac repair (81, 82). Transplantation of epicardial cells significantly drives the proliferative and regenerative capacity of cardiomyocytes, improving cardiac contractile function and therapeutic efficacy.

Moreover, cardiogenic follistatin-like 1 (Fstl1) in the heart stimulates cell cycle entry and cardiomyocyte division, which to some extent reverses cardiac remodeling after myocardial injury (83). Therefore, the precise delineation of cardiac progenitor cell fate determination and structural hierarchy will be an essential link in the study of cardiac development and disease treatment. In previous reports, cardiac progenitor cells expressing pluripotent Nkx2-5 and Isl1 transcription factors were reportedly actively involved in forming myocardial tissue, ECs, SMCs, and epicardium (84, 85). Since Wilms tumor 1(Wt1) and Tbx18 are strongly expressed as markers of the epicardium, it has been demonstrated that Nkx2-5 and Isl1 cardiac progenitors are involved in forming the epicardium during cardiac development. The Cre-loxP system was used to precisely track epicardium-specifically expressed transcription factors or markers, such as Wt1, Tbx18, Scx and Sema3D, TCF21, and GATA4/5. Genetic fate mapping was used to study the progeny development of epicardial progenitor cells, highlighting the remarkable contribution of cardiac precursors to the various cell lineages of the heart.

### Wt1^+^ proepicardial cell

As early as 1990, the Wt1 gene was believed to play multiple roles in cell proliferation, RNA metabolism, mesenchymal epithelium and tissue homeostasis (86, 87). As a tumor suppressor gene, Wt1 has long been used in the model of the Wt1-Cre system and is a reliable marker of the origin and fate of embryonic ECs (88). Using the transcription factor Wt1 to label cardiogenic precursors, gene tracing technology specifically tracks the final differentiation of cardiogenic progenitor cells into various components of the heart, including cardiomyocyte generation, diaphragm development, and EMT (89). During the past few years, studies have shown that epicardial cells have mainly originated from the extracardiac primordium and have played critical regulatory roles in embryonic myocardial differentiation and cardiovascular vasculature (90). Double-labeling of Rosa26fsLz and Z/Red as reporter genes, β-galactosidase (β-gal), or RFP under Cre recombinase induction accurately revealed that Wt1 was mainly concentrated in the epicardium and scattered in pericardial cells at E9.5-E15.5 (91). Notably, some Wt1-Cre-derived cells not only differentiated gradually into cardiomyocytes, but these cardiomyocytes also had contractile characteristics of kinetics, amplitude, and frequency. Moreover, the Wt1-Cre system is indispensable for studying epicardial cell fate. Efficient induction of Wt1-CreERT2 by TAM circumvents the binding of constitutive alleles to floxed structures early in cardiac development (92). Thus, identifying Wt1^+^ epicardial cells provides potential contributions and value to cardiac repair and regeneration potential.

### Tbx18^+^ epicardial cell

Epicardial progenitor cells expressing Tbx18 play critical regulatory roles in the early development and fate determination of coronary vessels, SMCs, and CFs. Gene knockout revealed that tbx18-deficient epicardium prevented cell differentiation and migratory behavior, causing developmental defects in the coronary vascular structure and function (93, 94). Lineage tracing was used to fully monitor the properties and hierarchy of the epicardium during development. Under the labeling of epicardial cells transcription factor Tbx18, the critical components of the heart were formed successively at embryonic stage E10.5, and were significantly localized near the inner wall of the ventricle and atrium. Next, by constructing Tbx18-CRE and R26-RLacZ mice, the fate of several types of cells in the embryonic heart was traced (95). Interestingly, the progeny of Tbx18-derived cells were not cardiomyocytes, but fibroblasts and vascular supporting cells. In adult cardiac lineage tracing analysis, Tbx18-labeled bulk progenitor cells differentiate into ventricular walls and atrioventricular valves but do not contain ECs.

However, Christoffels et al. suspect that the limitations and sensitivity of artificial expression tests and tools further lead to abnormal activation of Cre recombinase in the heart (96). Tbx18-labeled epicardial cells may not directly promote the formation of cardiomyocytes, but are expressed in the myocardium itself (97). Although Tbx18 knockout mice died after birth due to congenital defects, there were no significant differences in the atrioventricular structure and coronary vascular development during the embryonic period. This indirectly suggests that Tbx18 is not essential for epicardial development during the embryonic period. Notably, the co-expression of T-box protein-encoded transcription factor family members resulted in redundant effects. By constructing the R26-mTmG reporter gene and Tbx18-Cre mouse line, Notch and Tgfbr signaling pathways mediate the subversive expression of Tbx18VP16 in the epicardium and further affect the premature differentiation of SMCs (93). In addition, PCs are considered to possess the properties of mesenchymal stem cells (MSCs), but the differentiation of endogenous pericyte fate is controversial. PCs and vascular SMCs were tracked using the Tbx18-CreERT2 line (98). Genetic lineage tracing revealed that PCs and SMCs maintain their homogeneity in various pathological settings and do not have MSC characteristics.

### Scx and Sema3D contributions to coronary ECs

The proepicardial organ is a short-lived epicardial cell population that eventually migrates to the surface of the heart with most cells forming the epicardium. Scleraxis (Scx) and Semaphorin3D (Sema3D) are proepicardial markers, but there is heterogeneity in the Tbx18 and Wt1 expression domains (78, 99, 100). The contribution of the epicardium to coronary ECs remains controversial in previous studies. Since other subpopulations exist for the expression markers of Scx and Sema3D, the establishment of this subpopulation may yield a different differentiation fate than Tbx18 and Wt1. By crossing Scx-GFPCre and Sema3D-GFPCre mice with R26R-LacZ mice, lineage mapping revealed that pre-epicardial cells could give rise to coronary ECs.

### Essential role of TCF21, GATA4, and GATA5 in the epicardium

Transcription factor 21 (TCF21), a member of the class II basic helix–loop–helix (bHLH) family, is widely expressed in the epicardium of embryonic mesenchymal origin (101–103). In cardiac development, TCF21 activates epicardium-specific fate differentiation by regulating EMT. TCF21-LacZ and inducible TCF21-iCre mice with reporter genes R26R-LacZ, R26RYFP, and R26R-tdTomato mice were used for spatiotemporal gene function analysis and lineage tracing in the heart (104, 105). Studies have shown that TCF21 promotes the development of CFs and perivascular cells, but contributes little to cardiomyocytes and vascular SMCs. Therefore, genetic fate mapping is beneficial for revealing the regulatory mechanism of TCF21 on cardiac development and CVDs.

GATA4 (106) and GATA5 (107) are essential for epicardium formation and regulate cardiac development and coronary angiogenesis. Although it has been reported that the embryonic epicardium makes a negligible contribution to coronary ECs, the development of lineage tracing techniques may negate this conclusion. The emerging G2-Gata4-Cre and GATA5-Cre model mice were crossed with R26R-EYFP reporter mice to activate the expression of yellow fluorescent protein (108, 109). Genetic lineage tracing revealed that G2-GATA4 marks most of the epicardium at an early stage and is an integral part of coronary EC generation. In summary, multiple types of transcription factors are involved in the differentiation and maturation of the epicardium, which in turn regulates cardiac development and disease mechanisms.

## Genetic fate mapping defines ECS

When myocardial infarction occurs, normal coronary blood is blocked, and myocardial cells die in large numbers because they cannot get oxygen. In the process of heart repair, exploring the regeneration mechanism of coronary vessels is an important research field in the treatment of CVDs. Coronary vessels primarily develop from sinus endocardial cells (ECs), ventricular ECs, and epicardial cells (110, 111). In the early embryonic stage, endocardial cells form coronary ECs, and epicardial cells form vascular SMCs, both significantly contributing to angiogenesis (112–114). However, whether cardiac injury in adulthood leads to coronary angiogenesis remains a mystery. Ubil et al. (115) suggest that CFs take on ECs properties through mesenchymal endothelial cell transformation after cardiac injury, contributing to angiogenesis. However, using genetic lineage-tracing analysis, CFs were not further differentiated into ECs involved in angiogenesis (111). In 2017, Zhou Bin et al. generated a new genetic lineage-tracing system, using Npr3 as an EC marker, to extensively study the fate and origin of cardiac ECs throughout the developmental stages. Cardiac cushion MCs form as the early embryonic endocardium undergoes an endothelial-to-mesenchymal transition.

However, the specific differentiation mechanism of the endocardium is unclear. Based on the genetic lineage tracing performed by Cre recombinase, a dual-recombinase cross-targeting method was developed to improve the specificity of cell targeting (116). Fibroblasts, coronary parietal cells and adipocytes (ACs) in the heart are all derived from endocardial-derived cushion MCs, contributing to understanding the origin of cardiac development. ECs ultimately contribute to the coronary vessels of most of the heart. Lineage tracing and single-cell RNA sequencing in embryonic and adult mouse hearts uncover differential changes and plasticity in the transcriptional heterogeneity of coronary ECs (117). VEGF-B potently activates the ECs cycle *via* CreERT recombinase-mediated genetic cell lineage tracing (118). Endocardial-derived blood vessels develop toward the myocardium, promote the formation of coronary arteries, and improve the structure and remodeling of cardiac function. The widespread application of gene lineage tracing technology provides a strong guarantee of vascular reconstruction after myocardial infarction.

## Conclusion and future perspective

Genetic lineage tracing sheds light on the mysteries of developmental biology and facilitates deciphering the origin and fate of single-cell progeny. Widespread lineage-tracing systems have significantly advanced research in cardiac development, homeostasis, and tissue regeneration. To meet the requirements of heart development and disease prevention, Cre-loxP-based lineage-tracing technologies, such as dual recombinases, ProTracer, and MADM systems, are constantly innovating, improving the accuracy and stability of lineage-tracing. Tracking the development of epicardial and ECs reveals the components of each structural level of the heart, ruling out previous misconceptions. For example, epicardial cells substantially contribute to ventricular myocytes, whereas fibroblasts have little involvement in forming vascular ECs. The loss of cardiomyocytes after myocardial infarction is irreversible, exacerbating pathological remodeling and fibrosis of the heart. Supplementation of pre-existing cardiomyocytes is an effective means of cardiac regeneration. The lineage-tracing system uncovers the scientific question of the origin of cardiomyocytes and provides a reliable theoretical basis for the proliferation of cardiomyocytes. Moreover, it is controversial whether c-kit-expressing cardiac progenitors are the primary source of new cardiomyocytes. Lineage tracing has shown that c-kit cells proliferate cardiac ECs, but not cardiomyocytes (119, 120). TAM and DOX are generally used as time-controlled switches for inducible Cre-loxP initiation. The efficiency of site-specific recombination still needs to be improved, especially for gene deletion. These emerging tracers dissect cell origin and fate more precisely, offering new therapeutic strategies for congenital diseases and cardiac regeneration.

The extensive application value of the Cre-loxp system is compelling, but has some obvious constraints. The specific expression of Cre recombinase in targeted cells guarantees the accuracy of lineage tracing. Unfortunately, Cre recombinase expression in non-target cells may complicate lineage tracing, confusing tagged progeny cells. After the generation of mouse alleles and transgenic lines, the involvement of foreign genes may lead to tortuous structures of related genes, which may affect the functional expression of normal genes. Therefore, the accuracy of cell-lineage tracing is significantly compromised. Developing more recombination sites and tracers is an effective strategy for circumventing untargeted DNA site-specific recombination. Moreover, to enhance the recombination efficiency of inducible Cre-loxP, a self-cleavage-inducible CreER (sCreER) (121) came into being. Compared with traditional Cre-loxP, sCreER induces gene recombination more efficiently in ECs or fibroblasts. The iteration and innovation of genetic lineage tracing will provide further contributions and technical support for CVDs.

## Author contributions

TW is mainly responsible for drafting the manuscript and organizing the graphics. XC, KaW, JJ, XY, and SW assisted in drafting the manuscript and design of ideas. CL and KuW reviewed and revised the final manuscript. All authors contributed to the article and approved the submitted version.

## Funding

This work was supported by grant from the National Natural Science Foundation of China (81870236, 82070313, and 81770275), Taishan Scholar Program of Shandong Province, Major Research Program of the National Natural Science Foundation of China (No. 91849209), and Qingdao Scientific Program (No. 18–6-1-63-nsh).

## Conflict of interest

The authors declare that the research was conducted in the absence of any commercial or financial relationships that could be construed as a potential conflict of interest.

## Publisher’s note

All claims expressed in this article are solely those of the authors and do not necessarily represent those of their affiliated organizations, or those of the publisher, the editors and the reviewers. Any product that may be evaluated in this article, or claim that may be made by its manufacturer, is not guaranteed or endorsed by the publisher.
